# Spatial and temporal variation of malaria incidence in children under 10 years in a pyrethroid-resistant vector area in southern Benin

**DOI:** 10.1186/s12936-025-05353-2

**Published:** 2025-05-18

**Authors:** Edouard Dangbenon, Mintodê Nicodème Atchadé, Martin C. Akogbéto, Mahouton N. Hounkonnou, Landry Assongba, Hilaire Akpovi, Manisha A. Kulkarni, Natacha Protopopoff, Jackie Cook, Manfred Accrombessi

**Affiliations:** 1 Centre de Recherche Entomologique de Cotonou, Cotonou, Benin; 2 Institut Pierre Richet (IPR)/Institut National de Santé Publique (INSP), Bouaké, Côte d’Ivoire; 3 International Chair in Mathematical Physics and Applications (ICMPA-UNESCO Chair), University of Abomey-Calavi, 072BP50 Cotonou, Republic of Benin; 4 National Higher School of Mathematical Engineering and Modeling, Technologies, Engineering and Mathematics UNSTIM, National University of Sciences, Abomey, Benin; 5 Faculté Des Sciences Et Techniques, Université d’Abomey-Calavi, Abomey-Calavi, Benin; 6 Faculty of Infectious and Tropical Diseases, Disease Control Department, London School of Hygiene and Tropical Medicine, London, WC1E 7HT UK; 7 School of Epidemiology & Public Health, Faculty of Medicine, University of Ottawa, Ottawa, ON Canada; 8 Medical Research Council (MRC) International Statistics and Epidemiology Group, London School of Hygiene and Tropical Medicine, London, WC1E 7HT UK; 9 Malaria International Department, Population Services International (PSI), Cotonou, Benin

**Keywords:** Spatial and temporal heterogeneity, Malaria incidence, Hotspots, Benin

## Abstract

**Background:**

Spatial and temporal identification of malaria-endemic areas is a key component of vector-borne disease control. Strategies to target the most vulnerable populations, the periods of high transmission and the most affected geographical areas, should make vector-borne disease control and prevention programmes more cost-effective. The present study focuses on the spatial and temporal dynamics of malaria cases and the exogenous factors influencing the transmission in an area with pyrethroid-resistant mosquito vector populations.

**Methods:**

A prospective cohort study of 1806 children under 10 years of age was conducted over 20 months to assess the risk of malaria incidence in the Cove-Zagnanado-Ouinhi (CoZO) health zone located in southern Benin. Childhood malaria data were used to identify malaria hotspots according to months of follow-up using spatial scanning methods based on the Kulldoff algorithm. Stability scores were calculated by season to assess incidence heterogeneity. Incidence values by month were aggregated with meteorological data; and demographic data were merged to detect cross-correlation between incidence and meteorological variables. Generalized equation estimators were chosen for their ability to handle intra-group correlation, ensuring robust and interpretable results despite the complexity of the data to identify factors explaining the spatio-temporal heterogeneity of malaria incidence in the CoZO health zone.

**Results:**

Malaria incidence ranged from 1.41 (95% IC 0.96–2.08) to 13.91 (95% IC 12.22–15.84) cases per 100 child-months. Spatial heterogeneity in malaria transmission hotspots was observed over the study period, with relative risks ranging from 1.59 (p-value = 0.032) to 16.24 (p-value = 0.002). There was a significant negative association (correlation coefficient = − 0.56) between malaria incidence and temperature; and a slightly positive association (correlation coefficient = 0.58) between malaria incidence and rainfall. A significant association between malaria incidence with average house altitude (adjusted incidence rate ratio [aIRR] 1 (95% IC 0.99–1) P < 0.001), soil type aIRR 0.54 (0.39–0.75) p < 0.001 and temperature (incidence rate ratio [IRR] 0.69 (0.66–0.73) p < 0.001).

**Conclusion:**

This study uses innovative technologies such as remote sensing and geographic information systems (GIS) to analyse the environmental, meteorological and geographical factors influencing malaria transmission, thereby identifying high-risk areas and associated factors. It demonstrates that these tools improve the accuracy of control strategies, while highlighting the crucial role of the environment and human behaviour, paving the way for more targeted interventions against malaria and other vector-borne diseases.

**Supplementary Information:**

The online version contains supplementary material available at 10.1186/s12936-025-05353-2.

## Background

Despite the progress achieved in recent decades, malaria remains a major public health problem in sub-Saharan Africa (SSA). The World Health Organization (WHO) reported nearly 249 million cases in 2022, with 631,000 deaths in 85 endemic countries and areas [1]. Malaria control strategies mainly include use of insecticide-treated bed nets, indoor residual spraying, intermittent preventive treatment (IPT), and seasonal malaria chemoprevention [2]. The main factors associated with malaria-related deaths are often late diagnosis and failures to receive adequate treatments. Other major challenges include the spread of resistance in malaria vectors to insecticides and parasite resistance to anti-malarial drugs [3, 4].

In Benin, approximately 38,122 children under 5 years old die, 37.8% of them from malaria, diarrhoea and acute respiratory infections every year. The Zou department accounts for 47% of all deaths in Benin [5]. However, there are very few data available on the factors associated with malaria transmission in this area of Benin, as well as its spatial and temporal variations. Humidity, temperature and rainfall have been described as the common factors influencing malaria transmission in Benin [6–8]. Environmental and behavioural factors are also well-known to be associated with the spread of malaria [9]. Other factors that may influence transmission include vegetation, which can provide refuge and shelter for mosquitoes in adverse weather conditions, such as high summer temperatures, or impede mosquito movement [10–14]. The distribution of larval habitats and the development of immature mosquitoes may be influenced by topography and soil type, depending on water retention + time [15–17]. The local malaria transmission risk is increased by these larval habitats, as the mosquitoes born from them develop and reproduce nearby [18]. The distance between larval habitats also affects the spread of malaria [13, 19, 20]. In SSA, there are very few studies with larger sample sizes using small-scale meteorological data to investigate the malaria transmission. Malaria incidence is quite heterogeneous in southern Benin, but temporal variations are not well-understood, particularly in areas with a high prevalence of malaria vector resistance to standard insecticides. Additional evidence is, therefore, needed to better inform the spatial and temporal transmission of malaria incidence in malaria control strategies in low-income countries such as Benin.

The aim of this study was to identify and evaluate the meteorological, sociodemographic, epidemiological, entomological and environmental factors obtained by fine scale remote sensing that can explain spatial and temporal variations in malaria incidence in a high-risk region of southern Benin. Knowledge of the spatio-temporal distribution of the malaria incidence at fine scales is now essential for improving malaria control interventions.

## Methods

### Study area

The study was conducted in the Cove Zagnanado and Ouinhi (CoZO) health area in the Zou department of Benin. The site is located 155 km north of Cotonou, the economic capital of Benin. It has a savannah climate with an average annual temperature of 27.1 °C and an average annual rainfall of 1003.4 mm. There are four seasons, two of which are rainy (from March to July and from October to November). The CoZO health area comprises 123 villages spread over 18 sub-districts in 3 districts. According to the 2019 census conducted as part of the New Nets project [21], the health zone had a population of 216,289 inhabitants, including 66,341 children under the age of 10. In terms of health infrastructure, the health zone has 15 district health centres, a district hospital, 2 outposts and an isolated maternity unit [22].

### Data source and population

This study was embedded in the New Nets cluster-randomised control trial, which aimed to evaluate the efficacy of two long-lasting insecticidal nets (LLINs) with dual insecticides [pyrethroid-chlorfenapyr (Py-CFP) and pyrethroid-pyriproxyfen (Py-PPF)] compared to the standard LLIN (pyrethroid-only) for the control of malaria transmitted by pyrethroid-resistant vectors. The cluster-randomised controlled trial comprised 60 clusters with 20 each randomly assigned to 3 arms. The trial methodology has been fully described elsewhere [23]. A prospective cohort of 1805 children under 10 years, randomly selected from 60 clusters (30 children per cluster), was followed up for 20 months after the LLIN distribution.

### Data collection

Children were visited at home monthly during the dry season and twice a month during the rainy season. During these household visits, children with a temperature above 37.5 °C and/or a history of fever in the previous 48 h were systematically tested for malaria using a rapid diagnostic test (RDT). Positive children were treated with artemisinin-based combination therapy (ACT) according to the Ministry of Health guidelines. Data were collected using digital forms through the ODK (Open data kits) collect. The meteorological data were based on data downloaded from the Copernicus database “ERA5-Land monthly averaged data from 1950 to present” [24].

The cross-sectional baseline survey of the New Net project was also used to assess living standards by cluster. Household wealth data were used to calculate Social Economic Scores (SES), which were then split in two to create two categories of different living standards for different households. The modal class of these two categories is used to determine the standard of living of the cluster.

## Data processing and analysis

### Hotspot analysis

A spatial retrospective multiple analysis with point data organized in case files, population and GPS coordinates of child households according to months of intervention was used with the same parameter settings to standardize the analysis over each of the 20 months of the evaluation. The Kulldorff’s method with SaTScan V10.1.3 software [25, 26] is used, considering the maximum size of the default window (50% of the total population) while minimizing the number of cases to 2 with a statistical significance of less than or equal to 0.05. This method consists of an elliptical (circular) window of variable size, centre and rotation to group adjacent clusters into clusters of similar incidences. A Monte Carlo algorithm (9999 replicates) was used to test the Kulldorff statistic based on the likelihood ratio (Poisson model with purely spatial analysis). Only hot spots were considered in our analysis. Areas were classified as hotspots the malaria case incidence within the window was significantly higher than the incidence outside the window (p < 0.05). If at least one child in a study cluster was part of a significant cluster detected by SaTScan for a target period, this cluster was considered a hotspot.

### Stability analysis

Monthly maps of hotspot clusters were generated and stability scores were calculated based on the percentage of months in which a cluster was a hotspot [27, 28]. Stability maps of malaria hotspots using these percentages were generated for the study period, by season, study arm and soil type using ArcGis V10.3 software.

### Statistical analysis

Incidence data aggregated by cluster and month were used to find the statistical risk factor within R V4.3.2 software. The number of new malaria cases per cluster and the size of the dependent population were cumulated, along with aggregated meteorological data (temperature and rainfall), the soil type obtained by remote sensing [24], the average altitude of the children's houses and the standard of living of the clusters (obtained by principal component analysis of the assets available in households within the clusters) [21]. The stationarity of the obtained incidence and meteorological time series were determined using the Kwiatkowski-Phillips-Schmidt-Shin method. Cross-correlation functions were used to determine the lags between the stationary time series for incidence and each meteorological factor. The most significant lags near the origin were taken into account, as well as the value at the origin if it was significant at a threshold of 5%. The lag from the origin that is most correlated with incidence is included in the univariate and varied multiple analyses. Generalised estimating equations were then used, given their ability to handle grouped or correlated data (repeated measurements over time or spatial clusters) while providing robust parameter estimates to assess the impact of the different meteorological and environmental factors on malaria incidence. Following comparison of four different correlation structures (independent, autoregressive, unstructured, or exchangeable) using the QIC criterion (Quasi-likelihood under the Independence model Criterion), the autoregressive correlation structure was identified as the best fit to the data. Correction parameters were introduced to the models to correct for overdispersion. The impact test of the latter indicates that it has no significant impact. In order to address theoretical considerations such as the introduction of collinearity, which would render the estimates unstable, the performance of the models with the original meteorological variables was compared to models incorporating lagged variables and their dynamics, in order to select the final model. The robustness of the results is confirmed by the Bootstrap method.

### Ethical statement

This study was conducted in compliance with the highest ethical standards, guaranteeing the protection of the rights, dignity and well-being of the participants. It was approved by two recognized ethics committees: the Ethics Committee of the Ministry of Health of Benin (Reference N°6/30/MS/DC/SGM/DRFMT/CNERS/SA) and the Institutional Ethics Committee of the London School of Hygiene and Tropical Medicine (N°16237). These approvals attest that the study was designed and implemented in accordance with international ethical principles, in particular those set out in the Declaration of Helsinki.

Participation in this study was entirely voluntary, meaning that participants or their legal representatives had the right to refuse to participate or to withdraw at any time without incurring negative consequences. To ensure that participants were fully informed of the objectives, procedures, potential benefits and possible risks of the study, written informed consent was obtained from an adult guardian in the household or given by the participant if over 18 years of age. Consent was obtained for children over 10 years of age.

The study was conducted with particular attention to fundamental ethical principles, including respect for persons, beneficence (maximizing benefits and minimizing risks) and justice (fairness in the selection of participants). Informed consent procedures were tailored to the age and capacity of participants, ensuring that their rights and well-being were protected at every stage of the study.

## Results

In July 2020, 2283 households were randomly selected to form the base sample for the enrolment of children for the post-intervention cohort follow-up. There were 3129 eligible children, of whom 1829 were randomly selected and 1806 gave their consent, with 52.2% children under 5 years of age and a sex ratio of 1.07. In the study area, 36.7% of the surface was fine medium soil (Table 1).

**Table 1:** Baseline characteristics of the children enrolled in the study at the recruitment

Characteristics	All	Py-PPF-LLIN	Py-CFP-LLIN	Standard Py-LLIN
Number of clusters	60	20	20	20
Number of children	1806	604	601	601
Proportion of female children	868/1806 (48.1%)	285/604 (47.2%)	291/601 (48.4%)	293/601 (48.8%)
Proportion of children under 5 years	942/1806 (52.2%)	316/604 (52.3%)	304/601 (50.6%)	322/601 (53.6%)
Proportion of < 50% household with high SL)	32/60 (53.3%)	12/20 (60.0%)	10/20 (50.0%)	10/20 (50.0%)
Net usage the night before	1787/1806 (98.9%)	598/604 (99.0%)	593/601 (98.7%)	596/601 (99.2%)

Py-PPF: pyrethroid-pyriproxyfen; Py-CFP: pyrethroid-chlorfenapyr; SL: standard of living

Between August 2020 and March 2022, there were 2112 malaria cases in cohort children in the CoZo health zone (491, 732, and 889 cases in the Py-CFP LLIN arm, Py-PPF LLIN arm, and standard LLIN arm, respectively). The highest number of malaria cases (228) occurred in August 2021 and the lowest (23) in January 2022. The monthly average malaria incidence rate was 6.65 (95% CI 06.37–06.94) malaria cases per 100 child-month at risk for the period. The lowest malaria incidence rate of 01.41 (95% CI 00.96–02.08) cases per 100 child-month was reached in April 2021. This is likely to be due to the anti-malarial treatment administered to children in March 2021. In the CFP-LLIN arm, we observed a relatively low malaria incidence rate compared with the other arms, with an average of 04.61 (95% CI 04.22–05.03) cases per 100 child-month (Table S2-appendix).

**Fig. 1:**
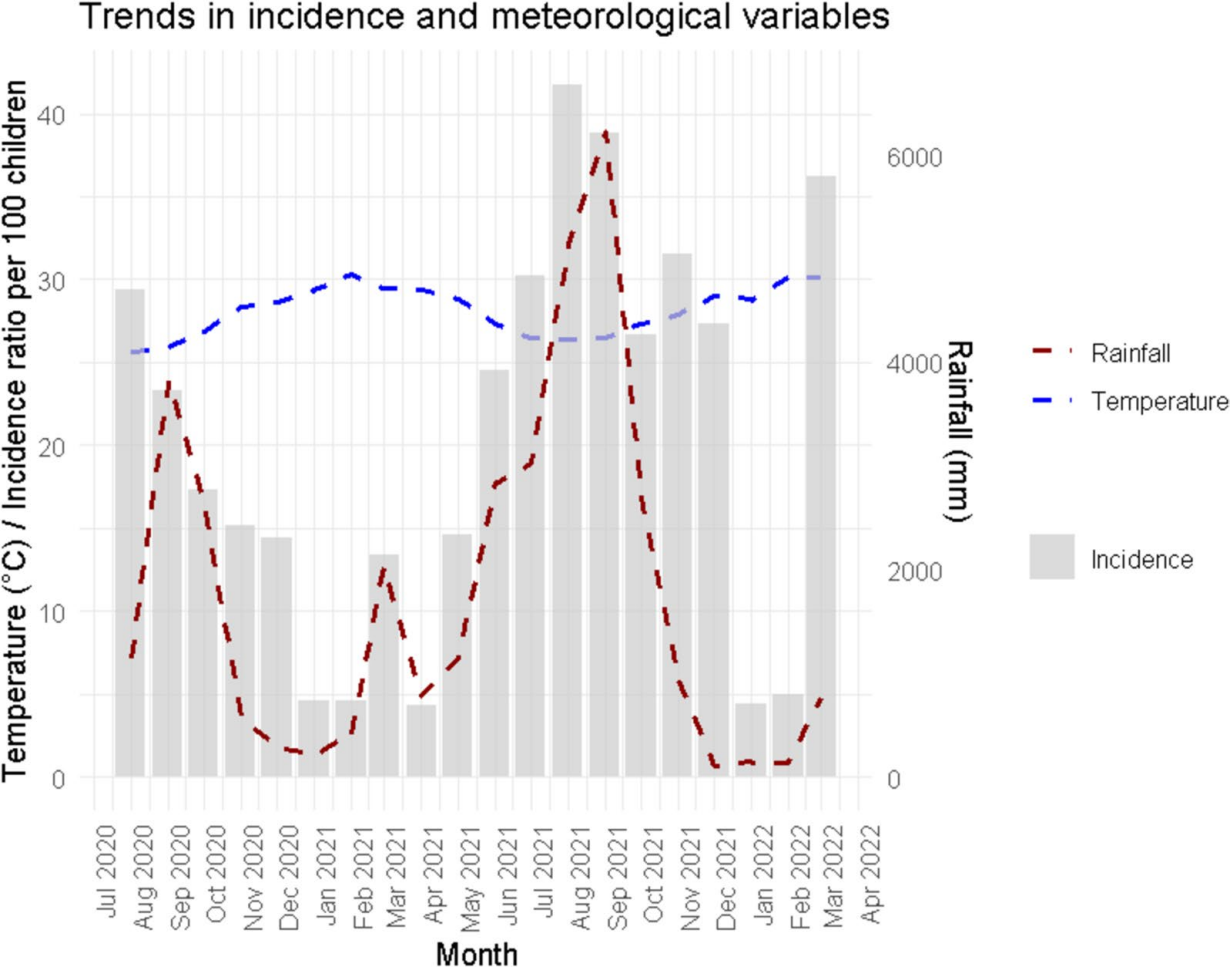
Trends in incidence and meteorological variables

Figure 1 shows a connection between malaria incidence and climatic conditions (temperature and rainfall). Incidence peaks often occur during periods of heavy rainfall following heat waves.

### Hotspot analysis

**Fig. 2:**
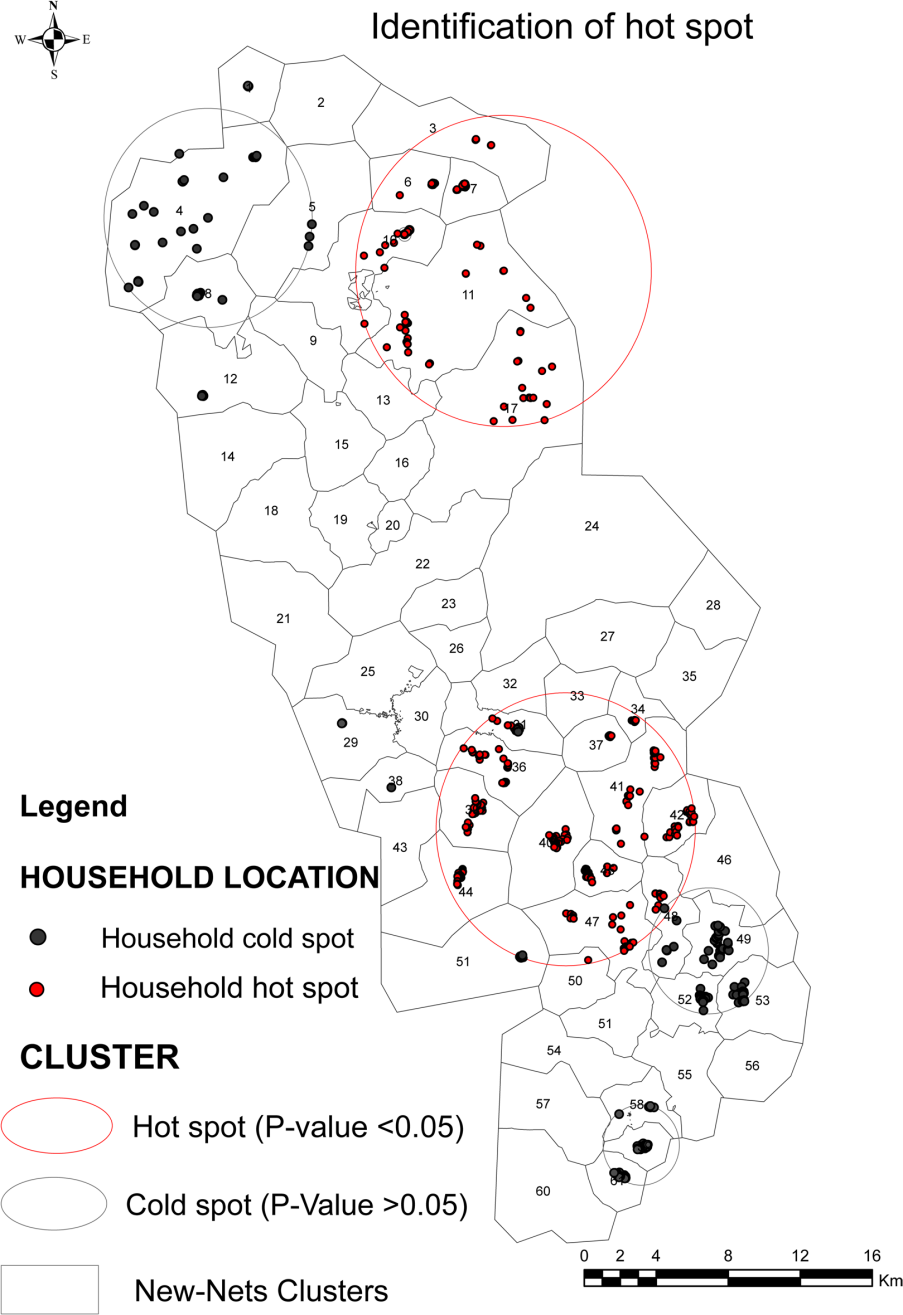
Identification of hot spots (The background map shows the survey area divided into 61 clusters. Cluster 56 is excluded from the New Nets pilot surveys. The Kulldoff scanning method identifies clusters of malaria incidence with 95% confidence. These clusters are shown as circles. A hotspot is indicated by a red circle. If there is at least one red-marked child’s home in a survey cluster in this hot spot, it is considered a hot cluster. Cold zones are indicated by nothing or by black circles with the child's home in black)

Malaria incidence-related maps, classified by cluster and month, were used to identify the hotspots of households most affected by malaria in the study region. Thirteen significant hotspots (P-value < 0.05) out of a total of 113 malaria-susceptible hotspots were observed over the whole study period (Fig. 2).

Malaria incidence in the significant hotspots varied between 0.86 and 11.37 cases per 100 child-months, with a relative risk (RR) of 6.05 (P-value < 0.001) and 7.78 (P-value = 0.05) by the estimated risk outside the cluster, respectively (Table S1-appendix).

### Stability analysis

#### Heterogeneity by season and by trial arm

**Fig. 3:**
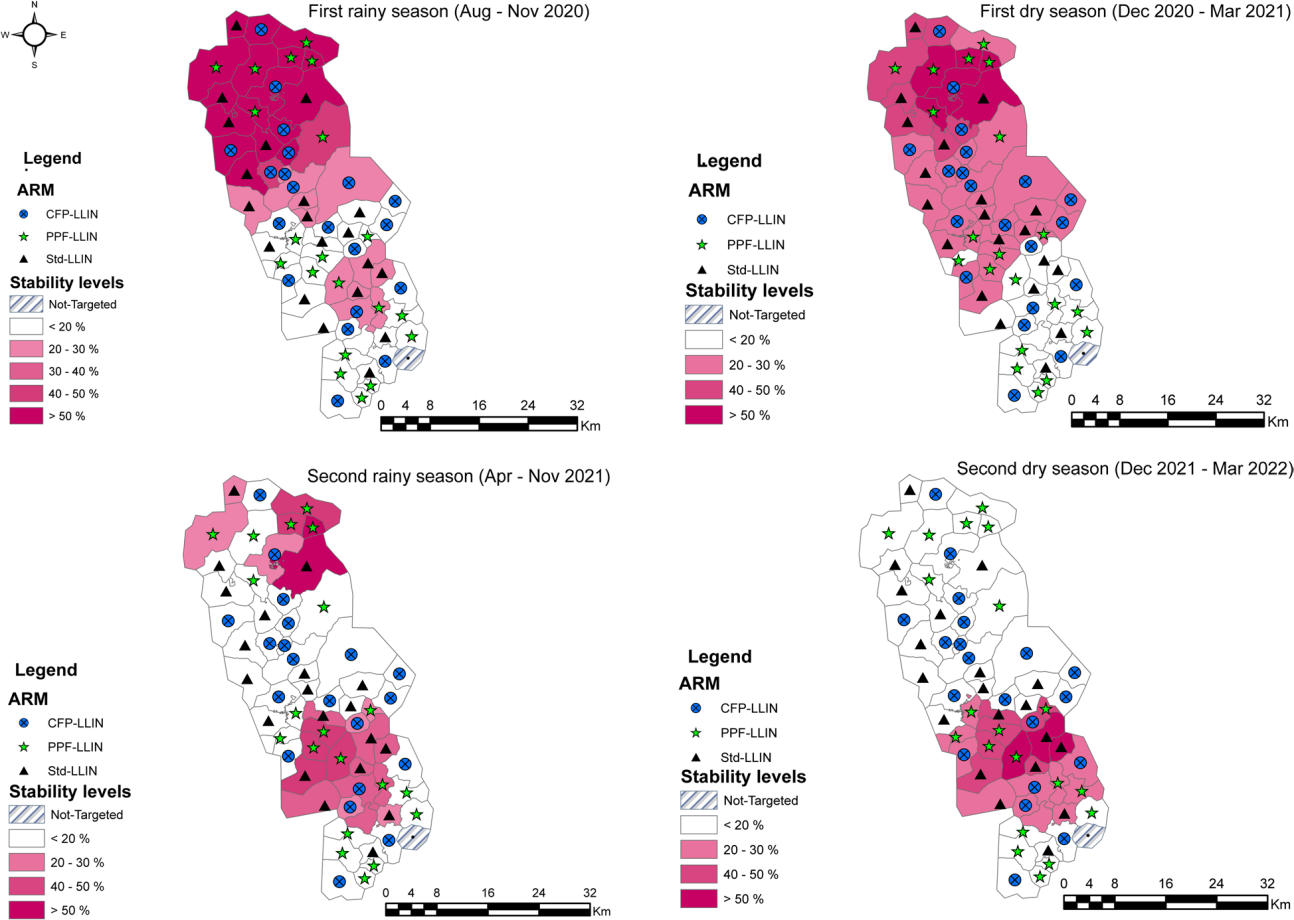
Stability of incidence by seasons and by trial arm (The four panels show stability maps using monthly incidence data by season and study arm. The presentation of each cluster is based on the number of months it was identified as a hotspot during a season. For example, clusters with a stability score above 50% indicate areas where incidence remains consistently high for at least half the months of in the season.)

Stability analysis of the significant clusters showed that the northern clusters were the most malaria-prone, with a malaria stability score of at least 20%, compared with some of the southern clusters, which maintained a stability score of less than 20% throughout the study period. Variability in levels of stability was also observed across seasons. There was a decrease in the number of clusters with a stability score of at least 20% between the first and second rainy and dry seasons. There was a change in the areas showing stability in malaria incidence between the two dry seasons (Fig. 3). Similarly, northern clusters showed high stability (> 50%) in malaria incidence during the two rainy seasons and the first dry season. In addition, the degree of stability in the incidence of malaria was distributed across the study arms. Most clusters using Py-CFP LLIN nets had a stability score below 30%, regardless of the season.

#### Heterogeneity by season and soil type

**Fig. 4:**
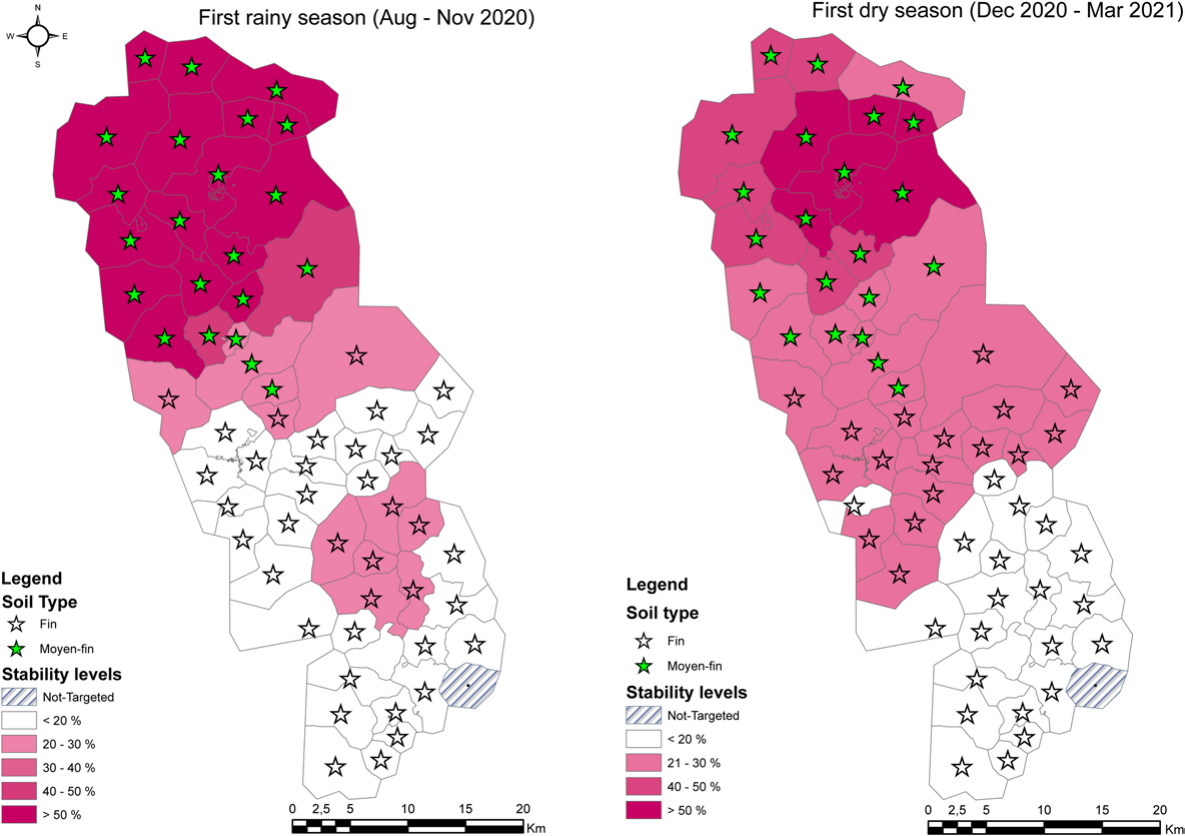
Stability of incidence by seasons and soil type (The level of stability in the early seasons is superimposed on the soil type. There are two soil types in our study area (medium-fine and fine). Depending on the season, the zone with the medium-fine soil type has an incidence stability of about 50%, while the zone with the fine soil type has an incidence stability of no more than 20%, regardless of the season)

Evaluation of the stability score by soil type shows a variation in the score depending on the soil type in the cluster. Clusters with the same soil type have almost the same characteristics in terms of malaria incidence stability. Clusters with the same geographical characteristics (soil type = medium fine) tend to have a high incidence area, regardless of the season. The opposite effect is observed in clusters geographically differentiated by fine soil type (Fig. 4).

### Temporal and risk factors analysis

**Fig. 5:**
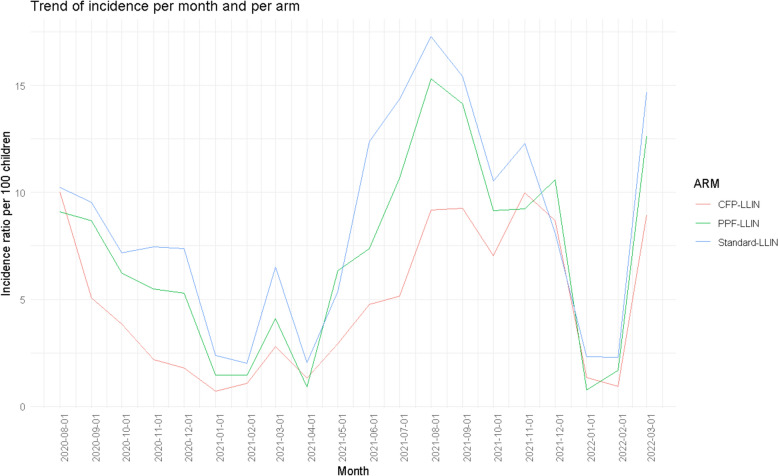
Trends in malaria incidence per month and trial arm among cohort children in the Cove Zagnanado and Ouinhi (CoZO) health area, Benin, 2020–2022

Regarding the type of LLIN used, clusters in the Py-CFP LLIN arm had the lowest incidence rates compared to the other arms, regardless of the month. However, similar trend in the incidence rate was observed in the other study LLIN groups (Py-PPF and standard LLIN groups) (Fig. 5).

**Fig. 6:**
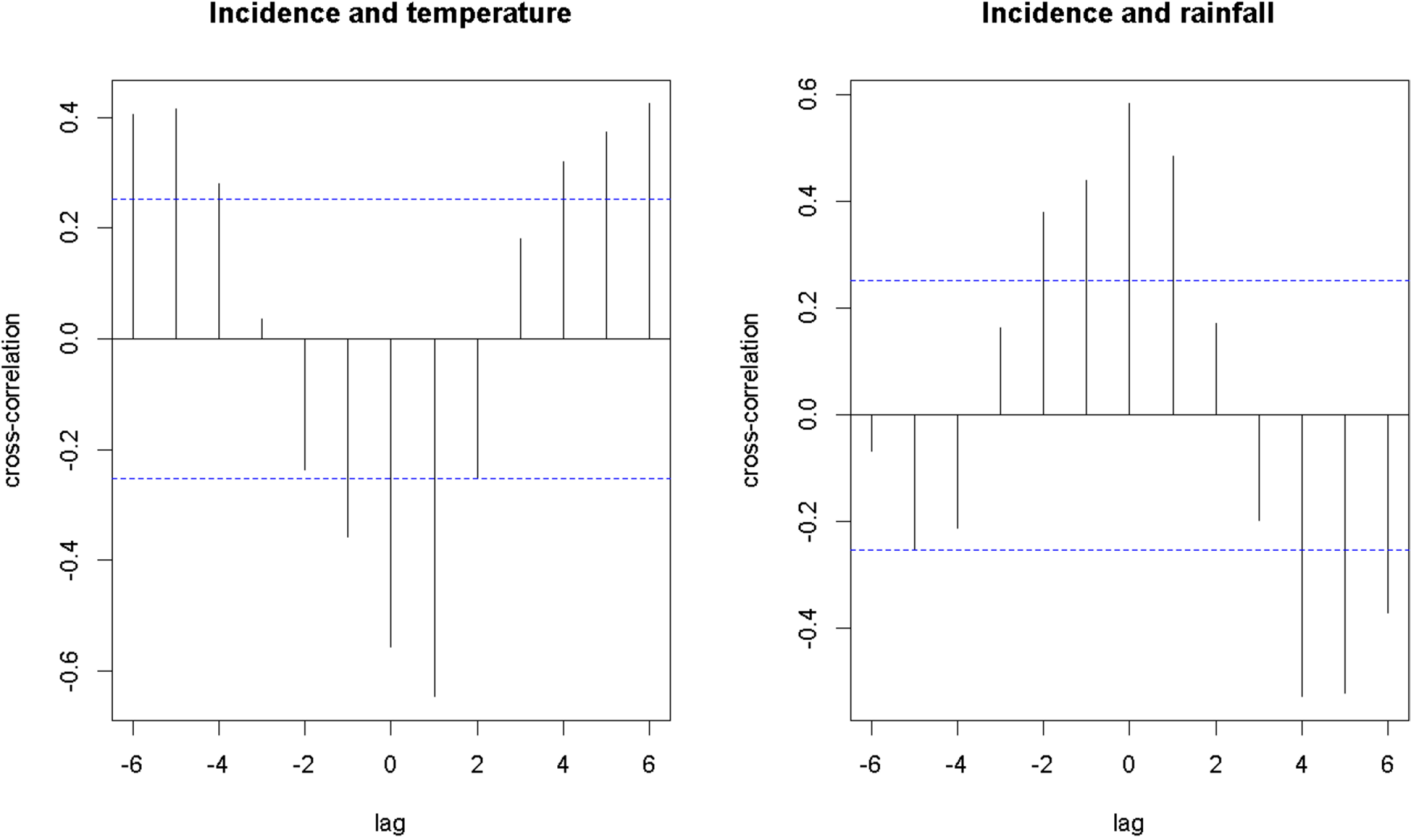
Cross-correlation function between malaria incidence, temperature and rainfall

Figure 6 presents the effect of meteorological variables on the average malaria incidence. A moderate positive correlation was observed between rainfall and malaria incidence (correlation coefficient = 0.58), while a moderate negative correlation was found with temperature (correlation coefficient = − 0.56). Significant shifts related to malaria incidence were observed with temperature (p < 0.001) and rainfall (p < 0.001). Significant lags of up to two months for rainfall and one month for temperature were identified. This means that rainfall in months t- 2, t- 1 and t are associated with incidence in month t. Similarly, the positive value of the Autocorrelation function (ACF) between incidence t and precipitation at date t- 1 suggests that the two series are moving in the same direction.

A significant association was found between malaria incidence and average house altitude, soil type, temperature, and type of bed net used (Table 2). The absolute effect of temperature on malaria incidence was 0.69, reaffirming the inverse relationship between temperature and malaria incidence. A 1 °C rise in temperature in the study area is associated with a 31% reduction in malaria incidence rate, and a 1 mm increase in rainfall is associated with a 9% increase in malaria incidence rate and a 14% increase in malaria incidence rate when all other variables remain constant. Ownership of bi-treated nets (Royal Guard^®^ LLIN (alpha-cypermethrin and pyriproxyfen) and Interceptor G2^®^ LLIN (alpha-cypermethrin and chlorfenapyr) was associated with a 44% and 23% reduction in malaria incidence rates, respectively, compared with ownership of Interceptor^®^ LLIN, a pyrethroid (alpha-cypermethrin) only LLIN.

**Table 2:** Factors associated with malaria incidence in cohort children living in the Cove Zagnanado and Ouinhi (CoZO) health area, Benin, 2020–2022

Variables		Univariate analysis		Multivariate analysis	
		IRR (IC 95%)	*P*-value	IRR* (IC 95%)	*P*-value
Soil type	Medium fine	Reference		Reference	
	Fine	0.78 (0.61–1)	0.048	0.54 (0.39–0.75)	p < 0.001
Standard of living	< 50% HH have a high SL	Reference		Reference	
	> 50% HH have a high SL	0.80 (0.62–1.04)	0.094	0.91 (0.70–1.20)	0.516
Study LLIN	Standard-LLIN	Reference		Reference	
	Py-PPF LLIN	0.81 (0.62–1.07)	0.15	0.77 (0.60–1)	0.048
	Py-CFP LLIN	0.54 (0.42–0.70)	p < 0.001	0.56 (0.45–0.71)	p < 0.001
Temperature (°)		0.69 (0.66–0.73)	p < 0.001	0.91 (0.84—0.98)	0.013
Rainfall (mm)		1.14 (1.12–1.15)	p < 0.001	1.09 (1.05–1.12)	P < 0.001
Altitude of Household		1 (1 − 1)	0.6	1 (0.99–1)	P < 0.001

HH: Household; SL: Standard of living, Py-PPF: pyrethroid-pyriproxyfen; Py-CFP: pyrethroid-chlorfenapyr

## Discussion

This study has highlighted the spatial and temporal variation of malaria incidence and the impact of meteorological factors in southern Benin, an area where malaria vectors are resistant to pyrethroid. There was a heterogeneous variability in malaria incidence on small scales, depending on several factors such as soil type, season, temperature, and rainfall. Spatial and temporal analysis or the identification of risk factors associated with malaria is a key component to better understanding malaria transmission and accelerating malaria control and elimination [29–32]. Some studies have shown the importance and impact of fine-scale results in informing national and local programme decisions regarding the control of waterborne, foodborne, vector-borne and other diseases [33, 34].

The study revealed a spatio-temporal variation in the distribution of malaria case hotspots. This variation depended on the season, or the type of bed net used. This suggests that malaria transmission is heterogeneous and confirms the findings that have identified a relationship between variation in malaria prevalence and use of mosquito nets (less than 17% malaria prevalence with insecticide-treated mosquito nets compared with no nets) and seasonal variation [35–39].

Meteorological factors (rainfall and temperature) had influenced malaria incidence. This finding has also been observed in many West and East African countries [40–43] where climatic conditions play a key role in disease transmission dynamics. This study also showed a negative significant effect of mean monthly temperature on malaria cases, which is consistent with a study conducted in Asembo-Siaya County, Kenya [44]. This could be due to increased mosquito mortality or reduced biting activity at extreme temperatures. These observations underline the importance of climatic factors in the spread of malaria and confirm their central role in modulating epidemiological dynamics. According to previous studies, rainfall and temperature play a crucial role in the spread of malaria vectors [45]. Above a certain threshold, they favour or hinder the reproduction of *Anopheles* mosquitoes, the vector of malaria transmission [46–48], as well as the developmental environment for larvae, the incubation period for parasites and the survival of mosquitoes [49, 50].

In this study, a negative effect of mean monthly temperature, suggesting that above a certain threshold, high temperatures can become unfavorable for malaria transmission. This can be explained by a reduction in mosquito survival or a too-rapid acceleration of the parasite’s development cycle, making transmission less efficient.

In addition to meteorological data, other factors can influence the risk of malaria transmission, such as the geography of the regions (altitude), seasonal variations (rainy or dry season), the environment (land cover), the standard of living and the type of bed nets. The results of study have confirmed the impact of these different factors on the malaria incidence. The effect of the average altitude of the houses where the children in the cohort live on the incidence of malaria is shown by studies conducted in northwestern Ethiopia and West Africa [51–53] where higher altitudes are associated with reduced malaria transmission. This is because *Anopheles* mosquitoes, the vectors of malaria, are less likely to survive and reproduce at higher altitudes due to lower temperatures and less favourable conditions. Environmental factors such as land cover and land use have a significant impact on the density and geographical distribution of malaria vectors.

No correlation was observed between living standards and malaria incidence, consistent with findings by [54, 55]. This result may be explained by the number of living standard classes we considered after our principal component analysis or by attributing the modal standard of living to the cluster, rather than an individual assessment of the components associated with malaria incidence. Modal allocation can lead to a loss of information, as it does not take into account the full distribution of living standards within clusters. Consequently, potential associations between specific living standards and malaria incidence may be overlooked. The previously cited studies may have used different approaches to measure and classify living standards, which could explain the divergent results. Standard of living may be more strongly correlated with access to health care or preventive measures, which is not necessarily the case in our study area as our children are followed up at home during the study period. Differences in living standards do not translate into significant differences in access to mosquito nets or anti-malarial treatment. However, housing conditions, such as the quality of dwellings and the presence of window screens, may also influence exposure to mosquito bites.

The use of insecticide-treated mosquito nets is one of the most effective interventions for reducing malaria transmission. The results confirmed that the type of net used had a significant impact on malaria incidence. Long-acting insecticide-treated nets (LLINs) are particularly effective in reducing mosquito bites and disease transmission. However, the effectiveness of nets also depends on their correct and regular use by the population. Awareness campaigns are therefore essential to maximize their impact.

The main limitation of our study is the lack of entomological data (collected at the local level) that could confirm and highlight the interactions between entomology, epidemiology, climatology and meteorology to better explain the variation in malaria incidence. Another limitation of this research is the failure to consider confounding factors such as human behaviour, including effective net use score, distance from water points to cluster, or malaria knowledge, which could affect malaria incidence.

## Conclusion

Knowledge of the distribution of malaria incidence rates at fine scales is essential for control interventions. The use of new technologies such as remote sensing (satellite imagery) and geographic information systems is enabling a better understanding of the environmental, meteorological and geographical factors that influence the distribution of vector-borne diseases. This study has enabled us to identify the factors associated with transmission risk and the epidemiological areas most at risk from malaria. Application of new tools such as remote sensing and GIS may improve malaria control strategies and better target intervention areas. The role of the environment and human behaviour is crucial in determining susceptibility to malaria transmission or vector-borne diseases.

## Supplementary Information

Supplementary Material 1

## Data Availability

No datasets were generated or analysed during the current study.

## Abbreviations

aIRR: Adjusted incidence rate ratio
ACF: Autocorrelation function
ACT: Artemisinin-based combination therapy
CI: Confidence interval
CoZO: Cove-zagnanado-ouinhi
GIS: Geographic information systems
HH: Household
HZ: Health zone
INC_100: Incidence per 100 children-month
IPT: Intermittent preventive treatment
IRR: Incidence rate ratio
LLINs: Long-lasting insecticidal nets
NC: Number of malaria new cases
NF: Exposure population
ODK: Open data kit
Py-CFP: Pyrethroid-chlorfenapyr
Py-PPF: Pyrethroid-pyriproxyfen
RDT: Rapid diagnostic test
RR: Relative risk
SL: Standard of living
sd: Standard deviation
WHO: World Health Organization
