# Supplementary material for: Spatial and temporal variation of malaria incidence in children under 10 years in a pyrethroid-resistant vector area in southern Benin
Source: Malar J. 2025 May 18;24:157. doi: 10.1186/s12936-025-05353-2 (PMC12087215; doi:10.1186/s12936-025-05353-2)
Supplement: Supplementary file 1 — Supplementary Material 1 [file 12936_2025_5353_MOESM1_ESM.docx]

**Table S1**. Description of hotspots

| **Order number** | **Month** | **Order number for monthly** | **Radius (Km)** | **Exposure population** | **Cases Observed** | **Cases Expected** | **Annual rate per 100,000** | **Incidence ratio per 100 children** | **Relative Risk** | **P-Value** |
| --- | --- | --- | --- | --- | --- | --- | --- | --- | --- | --- |
| 1 | Aug-20 | 1 | 28.32 | 149 | 70 | 43.4 | 46816.7 | 3.901391667 | 2.53 | 0.003 |
| 2 | Aug-20 | 2 | 0.46 | 5 | 6 | 1.36 | 128256.5 | 10.68804167 | 4.6 | 0.99 |
| 3 | Aug-20 | 3 | 0.011 | 0.6 | 2 | 0.16 | 357106.4 | 29.75886667 | 12.5 | 1 |
| 4 | sept-20 | 1 | 16.79 | 549 | 148 | 70.39 | 26922 | 2.2435 | 3.25 | p<0.001 |
| 5 | sept-20 | 2 | 0.1 | 4 | 5 | 0.54 | 117558.8 | 9.796566667 | 9.32 | 0.4437 |
| 6 | sept-20 | 3 | 0.15 | 8 | 6 | 1.06 | 72790.6 | 6.065883333 | 5.78 | 0.8595 |
| 7 | sept-20 | 4 | 0.023 | 2 | 3 | 0.25 | 154209.9 | 12.850825 | 12.16 | 0.975 |
| 8 | sept-20 | 5 | 0.033 | 0.7 | 2 | 0.089 | 289086.2 | 24.09051667 | 22.73 | 0.9946 |
| 9 | sept-20 | 6 | 0.093 | 5 | 4 | 0.6 | 86048 | 7.170666667 | 6.8 | 0.997 |
| 10 | oct-20 | 1 | 19.29 | 416 | 101 | 54.45 | 24255.8 | 2.021316667 | 2.52 | p<0.001 |
| 11 | oct-20 | 2 | 1.08 | 13 | 8 | 1.76 | 59342.7 | 4.945225 | 4.67 | 0.712 |
| 12 | oct-20 | 3 | 0 | 0.363 | 2 | 0.048 | 549355.7 | 45.77964167 | 42.37 | 0.838 |
| 13 | oct-20 | 4 | 0 | 0.46 | 2 | 0.06 | 433629.2 | 36.13576667 | 33.45 | 0.906 |
| 14 | oct-20 | 5 | 0.57 | 6 | 5 | 0.79 | 82842.6 | 6.90355 | 6.45 | 0.914 |
| 15 | oct-20 | 6 | 0.002 | 0.6 | 2 | 0.073 | 357106.4 | 29.75886667 | 27.54 | 0.976 |
| 16 | oct-20 | 7 | 0.2 | 2 | 3 | 0.31 | 126475.2 | 10.5396 | 9.79 | 0.996 |
| 17 | nov-20 | 1 | 11.94 | 212 | 58 | 30.39 | 27269.3 | 2.272441667 | 2.22 | 0.005 |
| 18 | nov-20 | 2 | 4.62 | 104 | 36 | 14.83 | 34684.7 | 2.890391667 | 2.7 | 0.005 |
| 19 | nov-20 | 3 | 4.3 | 49 | 18 | 7.08 | 36347.2 | 3.028933333 | 2.68 | 0.685 |
| 20 | nov-20 | 4 | 3.29 | 10 | 6 | 1.44 | 59323.9 | 4.943658333 | 4.24 | 0.998 |
| 21 | nov-20 | 5 | 0.047 | 5 | 4 | 0.65 | 87982.7 | 7.331891667 | 6.25 | 0.998 |
| 22 | nov-20 | 6 | 0.89 | 5 | 4 | 0.65 | 87950.4 | 7.3292 | 6.25 | 0.998 |
| 23 | dec-20 | 1 | 15.9 | 139 | 48 | 18.55 | 34525.4 | 2.877116667 | 3.29 | p<0.001 |
| 24 | dec-20 | 2 | 2.21 | 16 | 10 | 2.12 | 62975.2 | 5.247933333 | 4.97 | 0.174 |
| 25 | dec-20 | 3 | 0.05 | 2 | 4 | 0.26 | 202360.3 | 16.86335833 | 15.54 | 0.2816 |
| 26 | dec-20 | 4 | 0.18 | 2 | 3 | 0.25 | 162463.6 | 13.53863333 | 12.39 | 0.981 |
| 27 | dec-20 | 5 | 0.046 | 0.6 | 2 | 0.079 | 337267.2 | 28.1056 | 25.59 | 0.989 |
| 28 | dec-20 | 6 | 1.08 | 4 | 4 | 0.56 | 95526.9 | 7.960575 | 7.32 | 0.991 |
| 29 | dec-20 | 7 | 0.17 | 0.6 | 2 | 0.084 | 319516.3 | 26.62635833 | 24.24 | 0.992 |
| 30 | dec-20 | 8 | 0.033 | 0.7 | 2 | 0.088 | 303540.5 | 25.29504167 | 23.03 | 0.994 |
| 31 | janu-21 | 1 | 10.48 | 301 | 36 | 16.56 | 11930.6 | 0.994216667 | 2.99 | 0.006 |
| 32 | janu-21 | 2 | 0.57 | 8 | 4 | 0.45 | 48948.1 | 4.079008333 | 9.29 | 0.942 |
| 33 | janu-21 | 3 | 0.023 | 4 | 3 | 0.21 | 77830.9 | 6.485908333 | 14.65 | 0.947 |
| 34 | janu-21 | 4 | 0.11 | 1 | 2 | 0.063 | 173451.7 | 14.45430833 | 32.31 | 0.957 |
| 35 | janu-21 | 5 | 0.009 | 2 | 2 | 0.11 | 102718.2 | 8.55985 | 19.13 | 0.999 |
| 36 | febr-21 | 1 | 3.63 | 38 | 9 | 1.21 | 23610.6 | 1.96755 | 8.79 | 0.013 |
| 37 | febr-21 | 2 | 2.08 | 31 | 6 | 0.98 | 19457.7 | 1.621475 | 6.8 | 0.666 |
| 38 | febr-21 | 3 | 1.08 | 8 | 3 | 0.26 | 37320.6 | 3.11005 | 12.4 | 0.934 |
| 39 | febr-21 | 4 | 2.25 | 10 | 3 | 0.31 | 30868.5 | 2.572375 | 10.24 | 0.983 |
| 40 | febr-21 | 5 | 0.075 | 6 | 2 | 0.2 | 31292.8 | 2.607733333 | 10.19 | 0.999 |
| 41 | march-21 | 1 | 16.37 | 750 | 66 | 39.05 | 8783.8 | 0.731983333 | 3.38 | p<0.001 |
| 42 | march-21 | 2 | 0.26 | 6 | 4 | 0.29 | 71003.6 | 5.916966667 | 14.23 | 0.348 |
| 43 | april-21 | 1 | 0.15 | 15 | 7 | 0.48 | 47390.6 | 3.949216667 | 16.24 | 0.002 |
| 44 | april-21 | 2 | 0 | 0.39 | 2 | 0.013 | 505900.8 | 42.1584 | 159.7 | 0.103 |
| 45 | april-21 | 3 | 0.011 | 1 | 2 | 0.04 | 164075.9 | 13.67299167 | 51.77 | 0.679 |
| 46 | april-21 | 4 | 0.021 | 2 | 2 | 0.06 | 108407.3 | 9.033941667 | 34.19 | 0.917 |
| 47 | april-21 | 5 | 0.083 | 11 | 3 | 0.35 | 28280.2 | 2.356683333 | 9.03 | 0.995 |
| 48 | april-21 | 6 | 0.093 | 4 | 2 | 0.13 | 50172 | 4.181 | 15.81 | 0.999 |
| 49 | april-21 | 7 | 0.081 | 4 | 2 | 0.14 | 48181 | 4.015083333 | 15.18 | 0.999 |
| 50 | april-21 | 8 | 0.38 | 4 | 2 | 0.14 | 48142.2 | 4.01185 | 15.17 | 0.999 |
| 51 | may-21 | 1 | 3.52 | 47 | 26 | 6.24 | 55450.8 | 4.6209 | 4.6 | p<0.001 |
| 52 | may-21 | 2 | 6.51 | 162 | 51 | 21.56 | 31481.5 | 2.623458333 | 2.79 | p<0.001 |
| 53 | may-21 | 3 | 0.4 | 3 | 5 | 0.42 | 158010.4 | 13.16753333 | 12.13 | 0.157 |
| 54 | may-21 | 4 | 0.029 | 1 | 3 | 0.18 | 222102.8 | 18.50856667 | 16.91 | 0.778 |
| 55 | may-21 | 5 | 0 | 0.46 | 2 | 0.061 | 433629.3 | 36.135775 | 32.87 | 0.8911 |
| 56 | may-21 | 6 | 0.064 | 4 | 4 | 0.54 | 98712.4 | 8.226033333 | 7.54 | 0.984 |
| 57 | may-21 | 7 | 0 | 0.6 | 2 | 0.079 | 336318.4 | 28.02653333 | 25.49 | 0.985 |
| 58 | june-21 | 1 | 7.63 | 288 | 97 | 51.79 | 33594.6 | 2.79955 | 2.28 | p<0.001 |
| 59 | june-21 | 2 | 0.12 | 6 | 8 | 1.05 | 136383.8 | 11.36531667 | 7.78 | 0.05 |
| 60 | june-21 | 3 | 0.048 | 4 | 5 | 0.65 | 137845.7 | 11.48714167 | 7.78 | 0.677 |
| 61 | june-21 | 4 | 0.39 | 3 | 4 | 0.45 | 159651.5 | 13.30429167 | 9.01 | 0.913 |
| 62 | june-21 | 5 | 0.027 | 3 | 4 | 0.51 | 139477.4 | 11.62311667 | 7.87 | 0.973 |
| 63 | june-21 | 6 | 2.25 | 54 | 20 | 9.69 | 37014.8 | 3.084566667 | 2.14 | 0.996 |
| 64 | july-21 | 1 | 6.07 | 70 | 47 | 16.69 | 67091.6 | 5.590966667 | 3.07 | p<0.001 |
| 65 | july-21 | 2 | 5.41 | 32 | 23 | 7.53 | 72768.2 | 6.064016667 | 3.19 | 0.018 |
| 66 | july-21 | 3 | 11.04 | 516 | 165 | 123.08 | 31932 | 2.661 | 1.59 | 0.032 |
| 67 | july-21 | 4 | 0 | 0.46 | 2 | 0.11 | 433629.2 | 36.13576667 | 18.29 | 0.999 |
| 68 | aug-21 | 1 | 8.7 | 88 | 61 | 22.54 | 69121.4 | 5.760116667 | 3.02 | p<0.001 |
| 69 | aug-21 | 2 | 0.051 | 7 | 10 | 1.76 | 145199.4 | 12.09995 | 5.81 | 0.058 |
| 70 | aug-21 | 3 | 6.13 | 45 | 24 | 11.58 | 52911.9 | 4.409325 | 2.14 | 0.866 |
| 71 | aug-21 | 4 | 3.52 | 73 | 32 | 18.69 | 43719.9 | 3.643325 | 1.77 | 0.998 |
| 72 | aug-21 | 5 | 0.15 | 11 | 9 | 2.93 | 78467.5 | 6.538958333 | 3.12 | 0.998 |
| 73 | aug-21 | 6 | 0.13 | 10 | 8 | 2.51 | 81418 | 6.784833333 | 3.23 | 0.999 |
| 74 | aug-21 | 7 | 0 | 0.46 | 2 | 0.12 | 433629.3 | 36.135775 | 17.06 | 0.999 |
| 75 | sept-21 | 1 | 17.75 | 292 | 114 | 64.63 | 39018.5 | 3.251541667 | 2.12 | p<0.001 |
| 76 | sept-21 | 2 | 0.047 | 2 | 4 | 0.41 | 216031 | 18.00258333 | 9.87 | 0.818 |
| 77 | sept-21 | 3 | 1.07 | 4 | 5 | 0.9 | 123288.6 | 10.27405 | 5.64 | 0.987 |
| 78 | sept-21 | 4 | 3.51 | 49 | 22 | 10.93 | 44536.9 | 3.711408333 | 2.08 | 0.987 |
| 79 | sept-21 | 5 | 0.22 | 3 | 4 | 0.6 | 148068.5 | 12.33904167 | 6.76 | 0.995 |
| 80 | sept-21 | 6 | 0 | 0.46 | 2 | 0.1 | 433629.2 | 36.13576667 | 19.71 | 0.999 |
| 81 | sept-21 | 7 | 1.96 | 19 | 11 | 4.18 | 58240.3 | 4.853358333 | 2.68 | 0.999 |
| 82 | sept-21 | 8 | 0.87 | 3 | 4 | 0.69 | 128957.2 | 10.74643333 | 5.88 | 0.999 |
| 83 | oct-21 | 1 | 5.16 | 129 | 47 | 25.48 | 36486.8 | 3.040566667 | 1.99 | 0.178 |
| 84 | oct-21 | 2 | 0 | 0.1 | 2 | 0.02 | 1989919.5 | 165.826625 | 101.22 | 0.3128 |
| 85 | oct-21 | 3 | 0.24 | 7 | 7 | 1.32 | 104590.6 | 8.715883333 | 5.38 | 0.633 |
| 86 | oct-21 | 4 | 4.31 | 63 | 26 | 12.55 | 41001.7 | 3.416808333 | 2.17 | 0.751 |
| 87 | oct-21 | 5 | 1.79 | 6 | 6 | 1.13 | 105212.4 | 8.7677 | 5.4 | 0.942 |
| 88 | oct-21 | 6 | 0 | 0.295 | 2 | 0.059 | 674534.4 | 56.2112 | 34.31 | 0.949 |
| 89 | oct-21 | 7 | 0 | 0.46 | 2 | 0.091 | 433629.2 | 36.13576667 | 22.05 | 0.996 |
| 90 | oct-21 | 8 | 0 | 0.46 | 2 | 0.091 | 433629.2 | 36.13576667 | 22.05 | 0.996 |
| 91 | oct-21 | 9 | 0.025 | 2 | 3 | 0.31 | 189512.3 | 15.79269167 | 9.66 | 0.998 |
| 92 | oct-21 | 10 | 0.018 | 0.5 | 2 | 0.1 | 377032.3 | 31.41935833 | 19.17 | 0.999 |
| 93 | oct-21 | 11 | 0.14 | 2 | 3 | 0.35 | 168317 | 14.02641667 | 8.58 | 0.999 |
| 94 | nov-21 | 1 | 5.19 | 147 | 53 | 26.62 | 35990.5 | 2.999208333 | 2.22 | 0.008 |
| 95 | nov-21 | 2 | 0 | 0.135 | 2 | 0.024 | 1480121 | 123.3434167 | 82.46 | 0.385 |
| 96 | nov-21 | 3 | 3.58 | 42 | 19 | 7.62 | 45042.5 | 3.753541667 | 2.6 | 0.597 |
| 97 | nov-21 | 4 | 0 | 0.493 | 2 | 0.089 | 404720.6 | 33.72671667 | 22.54 | 0.993 |
| 98 | nov-21 | 5 | 0.37 | 2 | 3 | 0.31 | 175119.5 | 14.59329167 | 9.78 | 0.996 |
| 99 | dec-21 | 1 | 9.4 | 268 | 70 | 39.88 | 26033.9 | 2.169491667 | 2.42 | 0.001 |
| 100 | dec-21 | 2 | 0.42 | 0.8 | 2 | 0.11 | 263948.2 | 21.99568333 | 18.02 | 0.999 |
| 101 | dec-21 | 3 | 0.074 | 2 | 3 | 0.34 | 129994.5 | 10.832875 | 8.92 | 0.999 |
| 102 | dec-21 | 4 | 0 | 0.8 | 2 | 0.12 | 252950.4 | 21.0792 | 17.27 | 0.999 |
| 103 | janu-22 | 1 | 3.96 | 73 | 16 | 2.86 | 21975.3 | 1.831275 | 7.23 | p<0.001 |
| 104 | janu-22 | 2 | 1.59 | 12 | 4 | 0.47 | 33448 | 2.787333333 | 9.04 | 0.871 |
| 105 | janu-22 | 3 | 0.003 | 2 | 2 | 0.098 | 79879.1 | 6.656591667 | 20.98 | 0.987 |
| 106 | febr-22 | 1 | 5.47 | 164 | 17 | 4.3 | 10363.9 | 0.863658333 | 6.05 | 0.001 |
| 107 | febr-22 | 2 | 0.048 | 3 | 3 | 0.085 | 92920.5 | 7.743375 | 38.17 | 0.11 |
| 108 | febr-22 | 3 | 1.12 | 7 | 2 | 0.19 | 27346 | 2.278833333 | 10.92 | 0.999 |
| 109 | march-22 | 1 | 4.53 | 92 | 29 | 11.21 | 31482.5 | 2.623541667 | 2.88 | 0.017 |
| 110 | march-22 | 2 | 0.99 | 8 | 7 | 0.95 | 89276.6 | 7.439716667 | 7.58 | 0.133 |
| 111 | march-22 | 3 | 2.23 | 13 | 8 | 1.56 | 62264.7 | 5.188725 | 5.3 | 0.502 |
| 112 | march-22 | 4 | 2.53 | 43 | 15 | 5.25 | 34730 | 2.894166667 | 3.01 | 0.628 |
| 113 | march-22 | 5 | 0.47 | 16 | 8 | 1.91 | 51015.2 | 4.251266667 | 4.33 | 0.763 |

Table S2: *Description of monthly incidence ratio among 1,806 children, including at least 30 children aged between 6 months and 9 years per cluster.*

| Date | ALL | | Py-PPF LLIN arm | | Py-CFP LLIN arm | | Standard LLIN arm | |
| --- | --- | --- | --- | --- | --- | --- | --- | --- |
|  | Nc/NF | INC_100 (CI 95%) | Nc/NF | INC_100 (CI 95%) | Nc/NF | INC_100 (CI 95%) | Nc/NF | INC_100 (CI 95%) |
| 01/08/2020 | 33/335.83 | 09.83 (06.99 - 13.82) | 09/98.83 | 09.11 (04.74 - 17.50) | 12/119.83 | 10.01 (05.69 - 17.63) | 12/117.17 | 10.24 (05.82 - 18.03) |
| 01/09/2020 | 184/2387.56 | 07.71 (06.67 - 08.90) | 66/761.76 | 08.66 (06.81 - 11.03) | 42/829.02 | 05.07 (03.74 - 06.86) | 76/796.77 | 09.54 (07.62 - 11.94) |
| 01/10/2020 | 104/1808.34 | 05.75 (04.75 - 06.97) | 40/641.13 | 06.24 (04.58 - 08.51) | 23/595.71 | 03.86 (02.57 - 05.81) | 41/571.51 | 07.17 (05.28 - 09.74) |
| 01/11/2020 | 82/1631.43 | 05.03 (04.05 - 06.24) | 30/546.51 | 05.49 (03.84 - 07.85) | 12/548.19 | 02.19 (01.24 - 03.85) | 40/536.73 | 07.45 (05.47 - 10.16) |
| 01/12/2020 | 57/1172.06 | 04.86 (03.75 - 06.30) | 19/359.43 | 05.29 (03.37 - 08.29) | 07/391.87 | 01.79 (00.85 - 03.75) | 31/420.75 | 07.37 (05.18 - 10.48) |
| 01/01/2021 | 25/1641.52 | 01.52 (01.03 - 02.25) | 08/546.99 | 01.46 (00.73 - 02.92) | 04/548.57 | 00.73 (00.27 - 01.94) | 13/545.95 | 02.38 (01.38 - 04.10) |
| 01/02/2021 | 25/1650.36 | 01.51 (01.02 - 02.24) | 08/550.74 | 01.45 (00.73 - 02.90) | 06/551.63 | 01.09 (00.49 - 02.42) | 11/547.98 | 02.01 (01.11 - 03.62) |
| 01/03/2021 | 81/1817.61 | 04.46 (03.58 - 05.54) | 25/609.79 | 04.10 (02.77 - 06.07) | 17/608.97 | 02.79 (01.74 - 04.49) | 39/598.85 | 06.51 (04.76 - 08.91) |
| 01/04/2021 | 26/1835.27 | 01.42 (00.96 - 02.08) | 06/649.97 | 00.92 (00.41 - 02.05) | 08/603.52 | 01.33 (00.66 - 02.65) | 12/581.77 | 02.06 (01.17 - 03.63) |
| 01/05/2021 | 80/1637.65 | 04.89 (03.92 - 06.08) | 35/551.26 | 06.35 (04.56 - 08.84) | 16/546.65 | 02.93 (01.79 - 04.78) | 29/539.74 | 05.37 (03.74 - 07.73) |
| 01/06/2021 | 148/1787.97 | 08.28 (07.05 - 09.72) | 42/568.93 | 07.38 (05.46 - 09.99) | 28/588.91 | 04.75 (03.28 - 06.89) | 78/630.13 | 12.38 (09.91 - 15.45) |
| 01/07/2021 | 169/1697.32 | 09.96 (08.56 - 11.58) | 61/570.93 | 10.68 (08.31 - 13.73) | 30/583.38 | 05.14 (03.60 - 07.35) | 78/543.01 | 14.36 (11.51 - 17.93) |
| 01/08/2021 | 228/1638.57 | 13.91 (12.22 - 15.84) | 85/555.48 | 15.30 (12.37 - 18.93) | 50/544.41 | 09.18 (06.96 - 12.12) | 93/538.67 | 17.26 (14.09 - 21.15) |
| 01/09/2021 | 218/1694.88 | 12.86 (11.26 - 14.69) | 81/572.60 | 14.15 (11.38 - 17.59) | 54/583.59 | 09.25 (07.09 - 12.08) | 83/538.69 | 15.41 (12.43 - 19.11) |
| 01/10/2021 | 146/1644.95 | 08.88 (07.55 - 10.44) | 52/568.60 | 09.15 (06.97 - 12.00) | 39/554.50 | 07.03 (05.14 - 09.67) | 55/521.85 | 10.54 (08.09 - 13.73) |
| 01/11/2021 | 173/1638.32 | 10.56 (09.10 - 12.26) | 48/520.30 | 09.23 (06.95 - 12.24) | 54/540.59 | 09.99 (07.65 - 13.04) | 71/577.43 | 12.30 (09.74 - 15.52) |
| 01/12/2021 | 94/1028.41 | 09.14 (07.48 - 11.19) | 37/349.24 | 10.59 (07.68 - 14.62) | 30/345.46 | 08.68 (06.07 - 12.42) | 27/333.71 | 08.09 (05.55 - 11.80) |
| 01/01/2022 | 23/1560.74 | 01.47 (00.98 - 02.22) | 04/524.81 | 00.76 (00.29 - 02.03) | 07/518.82 | 01.35 (0.64 - 02.83) | 12/517.11 | 02.32 (01.32 - 04.09) |
| 01/02/2022 | 26/1582.63 | 01.64 (01.12 - 02.41) | 09/535.33 | 01.68 (00.87 - 03.23) | 05/524.74 | 00.95 (00.40 - 02.29) | 12/522.57 | 02.30 (01.30 - 04.04) |
| 01/03/2022 | 190/1576.75 | 12.05 (10.45 - 13.89) | 67/531.08 | 12.62 (09.92 - 16.03) | 47/527.07 | 08.92 (06.70 - 11.87) | 76/518.59 | 14.66 (11.70 - 18.35) |
| ALL | 2112/31768.63 | 06.65 (06.37 - 06.94) | 732/10613.73 | 06.90 (06.41 - 07.41) | 491/10655.91 | 04.60 (04.22 - 05.03) | 889/10498.99 | 08.47 (07.93 - 09.04) |
| NC: Number of malaria new cases NF: Exposure Population INC_100: Incidence per 100 children-month | | | | | | | | |
